# Tumor-Induced Osteomalacia due to Sarcomatoid Non–Small Cell Lung Carcinoma Confounded by Drug-Induced Fanconi Syndrome

**DOI:** 10.1210/jcemcr/luae101

**Published:** 2024-05-30

**Authors:** Bassam AlHamer, Ajit Singh, Carmen Patrascu, Mona Al Mukaddam

**Affiliations:** University of Pennsylvania Health System, Pennsylvania Hospital Department of Internal Medicine, Philadelphia, PA 19107, USA; University of Pennsylvania Health System, Pennsylvania Hospital Department of Internal Medicine, Philadelphia, PA 19107, USA; University of Pennsylvania Health System, Pennsylvania Hospital Nephrology, Philadelphia, PA 19107, USA; University of Pennsylvania Health System, Division of Endocrinology, Diabetes and Metabolism, Philadelphia, PA 19104, USA

**Keywords:** tumor-induced osteomalacia, hypophosphatemia, Fanconi, burosumab, fibroblast growth factor-23, selpercatinib

## Abstract

Tumor-induced osteomalacia (TIO) is an exceedingly rare paraneoplastic condition characterized by hypophosphatemia, osteomalacia, fragility fractures, and fatigue. A 39-year-old man was assessed for hemoptysis, pathological rib fractures, and fatigue, and was found to have a chest mass with lung metastasis. Biopsy of the mass suggested high-grade epithelioid and spindle cell neoplasm. He was initially treated for soft tissue sarcoma with an ifosfamide-based regimen and developed Fanconi syndrome that resolved on cessation of ifosfamide. Serum phosphate remained low. A low tubular maximum reabsorption of phosphate to glomerular filtration rate ratio (TmP/GFR) indicated disproportionate phosphaturia, while a severely elevated fibroblast growth factor-23 (FGF23) level enabled a diagnosis of TIO. He was started on phosphate and calcitriol supplementation. Subsequent next-generation sequencing demonstrated a *RET*-fusion mutation, leading to reclassification of his malignancy to a sarcomatoid non–small cell lung carcinoma. He was switched to selpercatinib, a targeted *RET*-kinase inhibitor approved for locally advanced or metastatic *RET*-fusion–positive solid tumors. This induced tumor remission with subsequent normalization of his FGF23 levels and hypophosphatemia. Despite the presence of a confounding etiology like drug-induced Fanconi syndrome, persistence of hypophosphatemia should prompt a workup of TIO, especially in the presence of a tumor.

## Introduction

Tumor-induced osteomalacia (TIO) is an extremely rare paraneoplastic condition with growing recognition. It is characterized by overproduction of fibroblast growth factor-23 (FGF23), often by mesenchymal tumors. FGF23 inhibits sodium-phosphate cotransporters in the proximal convoluted tubule (PCT), decreasing renal phosphate reabsorption, and suppresses 1-α hydroxylation of 25-hydroxyvitamin D decreasing gastrointestinal phosphate absorption. These effects induce osteomalacia, often refractory to vitamin D and calcium supplementation. Due to its rarity, there are often significant diagnostic delays in TIO (1). The World Health Organization recognizes phosphaturic mesenchymal tumors, mixed connective tissue variant (PMT-MCTs) as distinct tumors, often associated with TIO through production of FGF23. These are often benign and difficult to localize, making the gold-standard curative treatment, surgical resection, challenging (2, 3).

## Case Presentation

**Figure 1.**
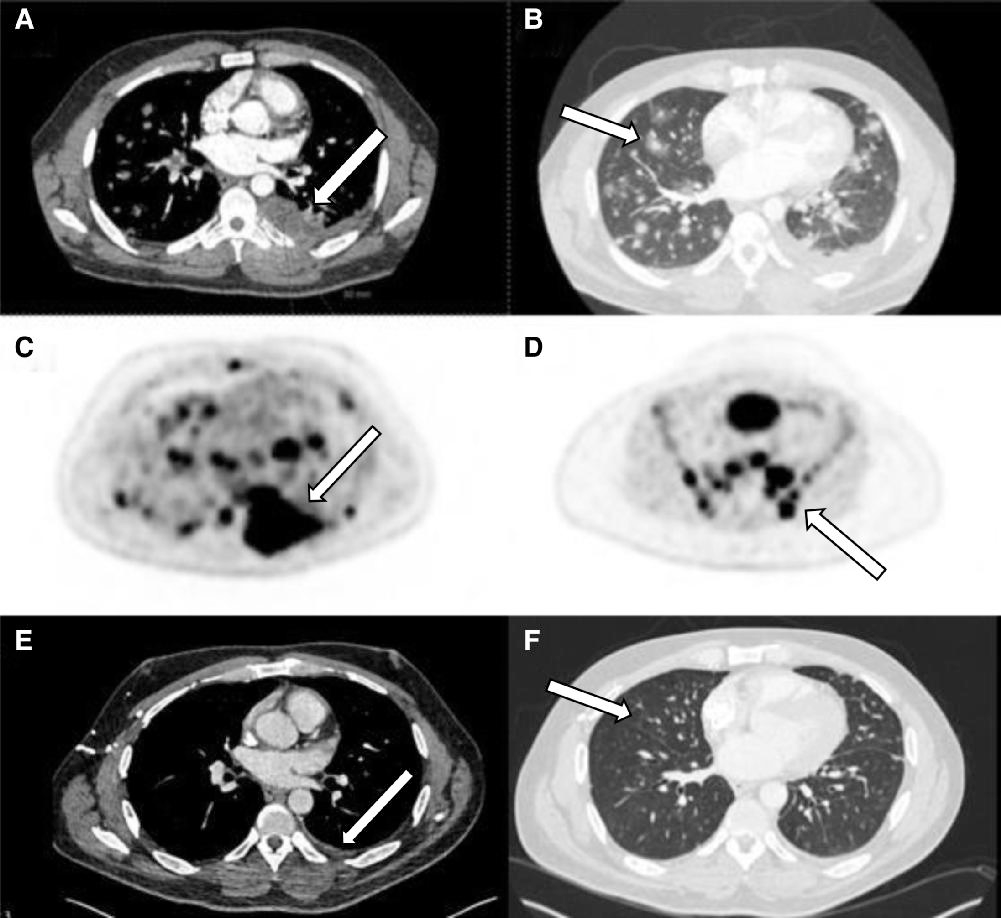
A, Arrow highlights a left pleural based bass, measuring 5.0 × 2.6 × 4.4 cm. B, Numerous lung nodules, some with central cavitation, with some associated ground-glass opacification. C, Arrow highlighting one such nodule. Fluorodeoxyglucose positron emission tomography computed tomography (FDG PET CT) redemonstrated a now enlarged 6.8 × 6.9-cm left pleural-based mass (highlighted by arrow), with an standardized uptake value (SUVmax) of 19.6. D, FDG PET CT also demonstrated extensive FDG-avid osseous lesions. One such representative lesion (arrow) included here on the left ischium with an SUVmax of 15.8. E, Marked improvement in size of pleural mass (arrow) and F, lung nodules compared to prior images 8 weeks after selpercatinib (arrow highlighting area of improvement of a lung nodule).

A 39-year-old man presented with hemoptysis on a background of pathologic rib fractures, bone-related pain, and persistent fatigue. Computed tomography with contrast (CT) showed a 6.8 × 6.9-cm left pleural–based mass and numerous pulmonary nodules suggesting metastatic disease. Fluorodeoxyglucose (FDG) positron emission tomography (PET)-CT demonstrated widespread hypermetabolic disease involving both lungs, lymph nodes, and bone. The pleural mass demonstrated a maximum standardized uptake value (SUVmax) of 19.6 (Fig. 1). He underwent CT-guided biopsy of that mass, which showed a high-grade epithelioid and spindle cell malignant neoplasm. The differential for that mass included high-grade sarcoma as well as other high-grade tumors such as thoracic SWI/SNF-related, matrix-associated, actin-dependent regulator of chromatin, subfamily A, member 4 (*SMARCA4*)-deficient undifferentiated tumors and sarcomatoid mesotheliomas (4).

Ten days after starting chemotherapy with AIM (adriamycin, ifosfamide, mesna), the patient was hospitalized with febrile neutropenia and pancytopenia that improved with antibiotics. During this admission, he developed multiple electrolyte disturbances (Table 1).

**Table 1.** Electrolyte disturbances at their nadir prior to cessation of ifosfamide, as well as hypoalbuminemia and a normal anion gap metabolic acidosis

Test	Worst value prior to cessation of ifosfamide
Serum phosphorus	<1.0 (2.4-7.7 mg/dL) <0.32 (0.77-1.52 mmol/L)
Serum sodium	129 (136-144 mEql/L) 129 (136-144 mmol/L)
Serum calcium	6.9 (8.9-10.3 mg/dL) 1.7 (2.2-2.6 mmol/L)
Serum potassium	3.1 (3.6-5.1 mEq/L) 3.1 (3.6-5.1 mmol/L)
Serum albumin	3.1 (3.5-5.1 g/dL) 31 (35-51 g/L)
Serum bicarbonate	18 (mEq/L) 18 (mmol/L)
Serum anion gap on day of lowest bicarbonate	8

## Diagnostic Assessment

A urinalysis showed significant new glucosuria without a history of diabetes. Given ongoing treatment with ifosfamide, severe electrolyte wasting and new glucosuria, he was diagnosed with ifosfamide-induced Fanconi syndrome. Chemotherapy was discontinued, and aggressive electrolyte supplementation was initiated. Most of his electrolyte derangements subsequently improved. Persistently low serum phosphate (<1 mg/dL, <0.32 mmol/L; normal 2.4-7.7 mg/dL, 0.77-1.52 mmol/L) with a normal parathyroid hormone (PTH) (43.8 pg/mL, 4.64 pmol/L; normal 15-65 pg/mL, 1.59-6.89 pmol/L) led to further investigation that revealed urine phosphate wasting with a low tubular maximum reabsorption of phosphate to glomerular filtration rate (TmP/GFR) (0.2848 mg/dL, 0.092 mmol/L; normal 3.09-4.18 mg/dL, 1.00-1.35 mmol/L), along with a low 1,25 OH vitamin D (18.1 pg/mL, 43.4 pmol/L; normal 19.9-79.3 pg/mL, 47.8-190.3 pmol/L) and a markedly elevated FGF23 (10 136 pg/mL, normal <56 pg/mL), confirming the diagnosis of TIO.

## Treatment

The patient was encouraged to maximize dietary intake of phosphate. He was also started on a regimen of intravenous (IV) and oral (PO) phosphate supplementation, as well as active vitamin D. Supplementation was increased over the coming weeks given his persistent hypophosphatemia, with frequent IV phosphate infusions required (Table 2). He was considered for initiation of burosumab-twza, a monoclonal antibody against FGF23, although this proved financially prohibitive. Subsequent next-generation sequencing tumor data returned several weeks later demonstrating an NCOA4 *RET*-fusion mutation. This suggested that his malignancy was consistent with a sarcomatoid non–small cell lung carcinoma (NSCLC). The decision was then made to treat him with selpercatinib, an oral *RET* tyrosine kinase inhibitor, resulting in remarkable improvement.

**Table 2.** Table of serum phosphate levels with corresponding elementary phosphorus, calcitriol and vitamin D supplementation

Day	Phosphate	Supplementation
0	<1.0 (2.4-7.7 mg/dL) <0.32 (0.77-1.52 mmol/L)	
3	1.2 (2.4-7.7 mg/dL) 0.39 (0.77-1.52 mmol/L)	
4	2.1 (2.4-7.7 mg/dL) 0.68 (0.77-1.52 mmol/L)	
5	2.1 (2.4-7.7 mg/dL) 0.68 (0.77-1.52 mmol/L)	-2 g twice daily of elemental phosphorus -50 000 units vitamin D weekly
∼4 wk	1.9 (2.4-7.7 mg/dL) 0.61 (0.77-1.52 mmol/L)	-1 g 3× daily of elemental phosphorus -50 000 units vitamin D weekly
∼5 wk	<1.0 (2.4-7.7 mg/dL) <0.32 (0.77-1.52 mmol/L)	-1 g 3× daily of elemental phosphorus -50 000 units vitamin D weekly
∼6 wk	1.4 (2.4-7.7 mg/dL) 0.45 (0.77-1.52 mmol/L)	-1.5 g 4× daily elemental phosphorus -50 000 units vitamin D weekly -0.5 µg of calcitriol daily
∼7 wk	<1.0 (2.4-7.7 mg/dL) <0.32 (0.77-1.52 mmol/L)	
∼8 wk	2.2 (2.4-7.7 mg/dL) 0.71 (0.77-1.52 mmol/L)	-1.5 g 4×daily elemental phosphorus -50 000 units vitamin D weekly -0.5 µg of calcitriol daily Selpercatinib started
∼14 wk	4.5 (2.4-7.7 mg/dL) 1.45 (0.77-1.52 mmol/L)	-Supplementation weaning ongoing
∼20 wk	4.3 (2.4-7.7 mg/dL) 1.39 (0.77-1.52 mmol/L)	-Off supplementation

Selpercatinib was started at around the 8-week point with subsequent normalization of serum phosphate.

## Outcome and Follow-up

The patient’s FGF23 levels decreased to 56 pg/mL within 12 weeks of starting selpercatinib therapy, coinciding with radiographic evidence of disease remission (see Fig. 1). Serum phosphate levels have since normalized, and he has not been hospitalized in more than 1 year (see Table 2). At 5 months, he developed a grade 3 transaminase elevation as defined by the Common Terminology Criteria for Adverse Events (CTCAE) version 5.0, without hyperbilirubinemia or coagulopathy. This resolved with pausing selpercatinib, and he has tolerated resumption at the same dose without recurrence.

## Discussion

Phosphate is required for a multitude of biochemical reactions in the human body. It forms part of the backbone of RNA and DNA, helps form cell membranes in the form of phospholipids, contributes to the creation of cellular energy through adenosine triphosphate, intermediates protein signaling through phosphorylation and dephosphorylation, and forms hydroxyapatite in bone (5).

**Figure 2.**
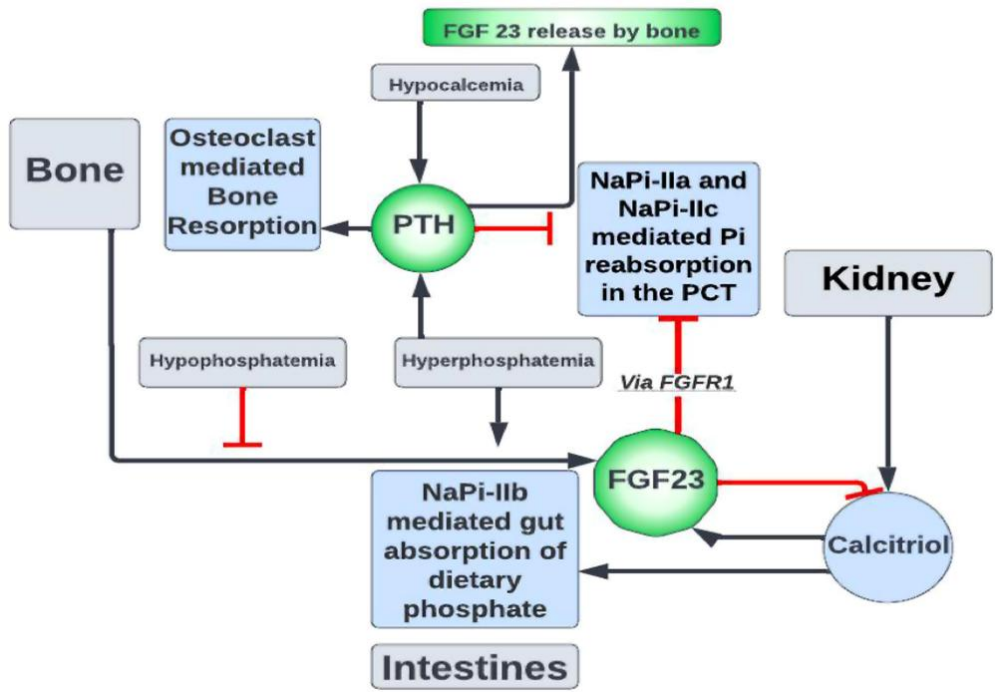
Phosphate is reabsorbed primarily in the proximal convoluted tubules by sodium-phosphate cotransporters (NaPi): NaPi-IIa and NaPi-IIc. Expression of these transporters is regulated by several hormones, including parathyroid hormone (PTH), calcitriol, and fibroblast growth factor (FGF) 23. Typically secreted by bone, FGF23 decreases NaPi expression, and therefore phosphate reabsorption, via binding of FGF receptor (FGFR) 1.

Phosphate metabolism is regulated by at least 4 major organ systems: The gastrointestinal tract, kidneys, bones, and soft tissue function under the influence of several hormones including PTH FGF23 and calcitriol (Fig. 2). Its movement is controlled by sodium-driven phosphate transporters (NaPi): NaPi-IIa, NaPi-IIb, NaPi-IIc, and PIT-2 (5).

Phosphate is absorbed in the gut by NaPi-IIb and is then freely filtered in the glomerulus before being almost 100% reabsorbed, mainly in the proximal tubule, via NaPi-IIa, NaPi-IIc, and PIT-2. Calcitriol causes increased gut absorption of phosphate (5). FGF23 is a strong stimulant of urinary phosphate excretion by binding to its receptor (FGFR) along with a cofactor necessary for signaling, alpha klotho, inducing proteolytic cleavage of NaPi-IIa and reducing phosphate reabsorption (5). It also indirectly decreases intestinal phosphate absorption by reducing activity of 1-α hydroxylase, thereby reducing levels of 1,25 dihydroxyvitamin D (5).

Renal causes of hypophosphatemia can be differentiated from other causes by measuring renal phosphate excretion (6). TmP/GFR is an accurate measure of the tubular threshold for phosphate excretion (6). It is essential when performing calculations to evaluate phosphaturia that the correct units be used in a consistent fashion. Tubular reabsorption of phosphate (%TRP) should be elevated in nonrenal cases of hypophosphatemia, representing the body's compensatory efforts (6). In cases of renal phosphate wasting, we may see a low %TRP, as was the case here. TmP/GFR adjusts for plasma phosphate and renal function and is thus seen as the most accurate measure of phosphate reabsorption (6). Like %TRP, it should be high in nonrenal cases of hypophosphatemia and was inappropriately low in our patient (Table 3). The differential for isolated phosphaturia includes TIO, hyperparathyroidism, X-linked hypophosphatemia, and Fanconi syndrome (proximal tubular wasting). Our patient's presentation later in life and lack of family history made a genetic cause unlikely.

**Table 3.** Fractional excretion of phosphate, tubular reabsorption of phosphate, and tubular maximum reabsorption of phosphate to glomerular filtration rate ratio

Measures of urinary phosphate excretion	Our patient	Normal
FePO_4_	≤71.5%	<20%
TRP	28.5%	85%-95%
TmP/GFR	0.285 mg/dL 0.092 mmol/L	3.09-4.18 mg/dL 1.00-1.35 mmol/L

These represent the gold standard of measuring urinary phosphate excretion. Calculations performed assuming serum phosphate of 1 mg/dL (0.32 mmol/L) as laboratory assays did not measure lower values.

Abbreviations: FePO_4_, fractional excretion of phosphate; TmP/GFR, tubular maximum reabsorption of phosphate to glomerular filtration rate ratio; TRP, tubular reabsorption of phosphate.

Fanconi syndrome represents dysfunction of the PCT's ability to reabsorb water, electrolytes, bicarbonate, glucose, and other solutes (7). Ifosfamide-induced Fanconi syndrome is thought to be at least partially mediated by a toxic metabolite, called chloroacetaldehyde (CAA) (8). Ifosfamide, a prodrug, is metabolized by cytochrome (CY) P450 3A4 and 2B6 into CAA. CAA then accumulates in the PCT, where it causes toxicity through depletion of adenosine triphosphate, generation of inflammatory cytokines, and increased oxidative stress (8). Risk factors for the development of Fanconi syndrome include higher total doses of ifosfamide, young age at treatment, and decreased renal mass (8). Persistence of hypophosphatemia after cessation of ifosfamide, in conjunction with resolution of his other electrolyte abnormalities, suggested an etiology beyond Fanconi syndrome as the cause of his hypophosphatemia. FGF23 elevation with renal phosphate wasting that did not improve despite cessation of ifosfamide, in the context of a new tumor, suggested a diagnosis of TIO.

Treatment of TIO is challenging due to difficulty in localizing and resecting FGF23-producing tumors. There is often a substantial delay before a phosphaturic tumor is found, with a mean time between onset of symptoms to diagnosis of 2.9 years (9). Different imaging modalities can be used to locate culprit tumors. Functional imaging such as ^68^Ga-DOTATATE PET/CT has been shown to be sensitive and specific, though it can be limited by the presence of other areas of high uptake (10). Octreoscan-SPECT/CT and Ga-DOTA-SST PET/CT were assessed in a meta-analysis by Jiang et al (10) and found to outperform F-FDG PET/CT (9). Functional imaging, along with cross-sectional imaging, may provide a framework for tumor localization.

Once found, surgical resection can be curative. Sun et al (11) found that 80% (32/40) of their cohort achieved normalization of serum phosphate levels 1 year post surgical resection. En bloc surgical resection with wide margins is recommended to decrease the risk of local recurrence. Radiofrequency ablation is an emerging avenue of treatment that may be associated with shorter lengths of stay and morbidity, though it has not yet been used on a large scale and long-term data on recurrence are limited (12).

When these are not feasible, phosphate and calcitriol supplementation is critical. Minisola et al (2) recommend the use of neutral phosphate and activated vitamin D supplementation to reduce the symptom burden of TIO and potentially bridge to other therapies. They recommend 1 to 3 g daily of elemental phosphate, as well as 0.5 to 1 μg daily of calcitriol. It is important to monitor for complications of such heavy supplementation, including hyperparathyroidism, nephrolithiasis, and reduced renal function.

New targeted therapies have been shown to be of benefit. Burosumab is a human monoclonal antibody that inhibits FGF23 approved for treatment of TIO, first shown to have benefit in children with X-linked hypophosphatemia (13). It received approval on the strength of 2 recent phase 2 studies in the United States and Asia . Interim analysis demonstrated improvement in bone mineralization, increased fracture healing, higher rates of serum phosphate normalization, and improved patient pain scores (14, 15).

Another potential therapeutic target for TIO is infigratinib, an FGFR1 to 3 tyrosine kinase inhibitor (16). Hartley et al (16) describe its use in 4 TIOs; 2 due to nonresectable PMTs and 2 with nonlocalized tumors. Patients demonstrated biochemical improvement while on infigratinib, though these effects did not continue after discontinuation of the drug. Remission, both radiographic and biochemical, was not achieved after stopping infigratinib. Unfortunately, the study was discontinued early due to a higher-than-expected rate of ocular side effects and the aforementioned data indicating that research participants were unlikely to have permanent remission.

Although not indicated for the treatment of TIO, the initiation of selpercatinib for treatment of his *RET*-positive NSCLC benefited our patient. *RET*-fusion mutations can be found across cancer subtypes, primarily in thyroid cancers, but also in NSCLC, colorectal cancers, and sarcomas. The LIBRETTO-001 trial, along with updated data released in 2021 and 2022, found that the overall objective response rate to selpercatinib was around 66% (17). Treatment-related side effects were largely related to transaminase elevations and drug hypersensitivity, but only 2% of patients enrolled required discontinuation of the drug despite dose adjustment (17). Selpercatinib is now approved for the treatment of any widespread or metastatic solid cancer driven by a *RET* mutation.

To our knowledge, this is the first reported case of TIO in association with a *RET*-fusion–positive tumor and the first reported case in which it has been controlled with the use of selpercatinib. Like many paraneoplastic phenomena, it is likely that effective treatment of this *RET*-positive NSCLC with selpercatinib decreased secretion of FGF23, which led to normalization of the patient’s serum phosphate levels.

Staining for FGF23 receptors was not performed as part of the initial histopathological workup of his tumor, as there was no suspicion for TIO at the time. Treatment with burosumab was not initiated due to cost, with the need for treatment eventually dissipating due to improvement with selpercatinib.

## Learning Points

- TIO is an exceedingly rare paraneoplastic condition caused by overexpression of FGF23.
- Fanconi syndrome, like TIO, causes renal phosphate wasting. In this case, persistence of hypophosphatemia after resolution of Fanconi syndrome prompted further investigation leading to a diagnosis of TIO.
- Definitive treatment of TIO is removal of the offending tumor. When this is not possible, calcitriol and phosphate supplementation is critical. Burosumab has also been shown to be effective in the treatment of TIO.
- This case demonstrates TIO in association with a sarcomatoid non–small cell lung carcinoma, which has not been previously described in the literature.
- Given the presence of a *RET*-fusion mutation, selpercatinib was used for treatment of this malignancy resulting in remission as well as resolution of TIO.

## Data Availability

Data sharing is not applicable to this article as no data sets were generated or analyzed during the current study.

## Abbreviations

CAA: chloroacetaldehyde
CT: computed tomography
FDG: fluorodeoxyglucose
FePO_4_: fractional excretion of phosphate
FGF23: fibroblast growth factor-23
FGFR: fibroblast growth factor receptor
NaPi: sodium-driven phosphate transporters
NSCLC: non–small cell lung carcinoma
PCT: proximal convoluted tubule
PET: positron emission tomography
PMT: phosphaturic mesenchymal tumor
PTH: parathyroid hormone
SUVmax: maximum standardized uptake value
TIO: tumor-induced osteomalacia
TmP/GFR: tubular maximum reabsorption of phosphate to glomerular filtration rate ratio
TRP: tubular reabsorption of phosphate

## References

[1] Hidaka N, Koga M, Kimura S, et al Clinical challenges in diagnosis, tumor localization and treatment of tumor-induced osteomalacia: outcome of a retrospective surveillance. J Bone Miner Res. 2020;37(8):1479‐1488. 10.1002/jbmr.4620 35690913

[2] Minisola S, Fukumoto S, Xia W, et al Tumor-induced osteomalacia: a comprehensive review. Endocr Rev. 2023;44(2):323‐353. 36327295 10.1210/endrev/bnac026

[3] Bosman A, Palermo A, Vanderhulst J, et al Tumor-induced osteomalacia: a systematic clinical review of 895 cases. Calcif Tissue Int. 2022;111(4):367‐379. 35857061 10.1007/s00223-022-01005-8 PMC9474374

[4] Herpel E, Rieker RJ, Dienemann H, et al SMARCA4 and SMARCA2 deficiency in non–small cell lung cancer: immunohistochemical survey of 316 consecutive specimens. Ann Diagnos Pathol. 2017;26:47‐51. 10.1016/j.anndiagpath.2016.10.006 28038711

[5] Levi M, Gratton E, Forster IC, et al Mechanisms of phosphate transport. Nat Rev Nephrol. 2019;15(8):482‐500. 31168066 10.1038/s41581-019-0159-y

[6] Payne RB . Renal tubular reabsorption of phosphate (TmP/GFR): indications and interpretation. Ann Clin Biochem. 1998;35(Pt 2):201‐206. 9547891 10.1177/000456329803500203

[7] Klootwijk ED, Reichold M, Unwin RJ, Kleta R, Warth R, Bockenhauer D. Renal fanconi syndrome: taking a proximal look at the nephron. Nephrol Dial Transplant. 2015;30(9):1456‐1460. 25492894 10.1093/ndt/gfu377

[8] Hanly L, Chen N, Rieder M, Koren G. Ifosfamide nephrotoxicity in children: a mechanistic base for pharmacological prevention. Expert Opin Drug Saf. 2009;8(2):155‐168. 19309244 10.1517/14740330902808169

[9] Feng J, Jiang Y, Wang O, et al The diagnostic dilemma of tumor induced osteomalacia: a retrospective analysis of 144 cases. Endocr J. 2017;64(7):675‐683. 28450684 10.1507/endocrj.EJ16-0587

[10] Jiang Y, Hou G, Cheng W. Performance of 68Ga-DOTA-SST PET/CT, octreoscan SPECT/CT and 18F-FDG PET/CT in the detection of culprit tumors causing osteomalacia: a meta-analysis. Nucl Med Commun. 2020;41(4):370‐376. 32000173 10.1097/MNM.0000000000001163 PMC7077972

[11] Sun ZJ, Jin J, Qiu GX, Gao P, Liu Y. Surgical treatment of tumor-induced osteomalacia: a retrospective review of 40 cases with extremity tumors. BMC Musculoskelet Disord. 2015;16(1):43. 25879543 10.1186/s12891-015-0496-3 PMC4349610

[12] Horng JC, Van Eperen E, Tutton S, Singh R, Shaker JL, Wooldridge AN. Persistent phosphaturic mesenchymal tumor causing tumor-induced osteomalacia treated with image-guided ablation. Osteoporos Int. 2021;32(9):1895‐1898. 33655402 10.1007/s00198-020-05795-1

[13] Hartley IR, Collins MT. Burosumab for tumor-induced osteomalacia: not enough of a good thing. J Bone Miner Res. 2021;36(12):2453‐2454. 33950530 10.1002/jbmr.4318

[14] De Beur SMJ, Miller PD, Weber TJ, et al Burosumab for the treatment of tumor-induced osteomalacia. J Bone Miner Res. 2021;36(4):627‐635. 33338281 10.1002/jbmr.4233 PMC8247961

[15] Imanishi Y, Ito N, Rhee Y, et al Interim analysis of a phase 2 open-label trial assessing burosumab efficacy and safety in patients with tumor-induced osteomalacia. J Bone Miner Res. 2021;36(2):262‐270. 32967046 10.1002/jbmr.4184 PMC7988547

[16] Hartley IR, Roszko KL, Li X, et al Infigratinib Reduces Fibroblast Growth Factor 23 (FGF23) and Increases Blood Phosphate in Tumor-Induced Osteomalacia. JBMR Plus. 2022;6(8):e10661. 35991529 10.1002/jbm4.10661 PMC9382865

[17] Drilon A, Subbiah V, Gautschi O, et al Selpercatinib in patients with RET fusion-positive non-small-cell lung cancer: updated safety and efficacy from the registrational LIBRETTO-001 phase I/II trial. J Clin Oncol. 2023;41(2):385‐394. 36122315 10.1200/JCO.22.00393 PMC9839260

